# A Case of Plasma Cell Leukemia Arising De Novo: A Rare and Fatal Disease

**DOI:** 10.7759/cureus.71393

**Published:** 2024-10-13

**Authors:** Hisham B Sweidan, Abdullah Jarrah, Feng Zhu, Yating Wang

**Affiliations:** 1 Internal Medicine, Detroit Medical Center (DMC) Sinai Grace Hospital, Detroit, USA; 2 Internal Medicine, Detroit Medical Center (DMC) Sinai Grace Hospital, Wayne State University, Detroit, USA; 3 Hematology and Oncology, Ascension Providence Hospital, South Field, USA

**Keywords:** clinical hematology, cytology and hematology, hematology laboratory, malignant hematology, multiple myelomam, plasma cell leukaemia, practicing internal medicine

## Abstract

Plasma cell leukemia (PCL) is an aggressive form of multiple myeloma characterized by an increasing number of circulating plasma cells in the peripheral (circulating) blood. Primary PCL (pPCL) is the form of monoclonal gammopathy that is the most severe with a high mortality rate. Its incidence will be increasing, given the expected changes in clinical criteria for diagnosis. We present a case to help further understand the disease and the proposed criteria changes, as more research will be needed to better understand the impact of specific genetic findings on the prognosis of pPCL and secondary PCL (sPCL). We also feature the presentation, morphological changes in the peripheral smear, and cytogenetics of this rare disease.

## Introduction

Plasma cell leukemia (PCL) is a rare plasma cell disorder characterized by circulating plasma cells on peripheral blood smears in patients who meet the criteria for multiple myeloma (MM). It carries a poor prognosis with overall survival of only 11.3 months after diagnosis [1]. PCL is a rare hematological malignancy with a yearly incidence rate of 1:2,000,000 [2]. It occurs in 1-4% of patients with MM [3]. It is classified as primary plasma cell leukemia (pPCL), which arises de novo, and secondary plasma cell leukemia (sPCL), which arises mostly in patients with MM with plasma cells circulating in the peripheral smear. A study published recently in November 2022 reported in the Journal of Clinical Oncology that a level of >2% circulating tumor cells (CTCs) would define PCL-like multiple myeloma [4]. We present a case of PCL in a patient with no prior history of MM, featuring peripheral smear findings along with genetics studies to help better understand the disease.

## Case presentation

A 70-year-old African American patient with a prior history of alcohol and tobacco use presented to the hospital following a six-week history of generalized fatigue, weakness, anorexia, and weight loss. He reported increasing shortness of breath and night sweats one week before his presentation. He denied fever, chills, chest pain, wheezing, coughing, nausea and vomiting, abdominal pain, hematuria, melena, or hematochezia. He had smoked a pack a day since the age of 13 but quit five years before presentation. He drank alcohol heavily, almost 15-18 drinks daily. Physical examination revealed a tired-appearing male. His initial vitals showed blood pressure(BP) of 120/59 mmHg, heart rate of 82 bpm, O2 saturation of 94%, and respiratory rate of 20 breaths per minute. Lung auscultations were clear, with no crackles or wheezing. Heart auscultation revealed a normal S1 and S2, and no edema of lower extremities. Abdominal examination revealed some generalized tenderness.

Initial laboratory studies revealed a white blood count (WBC) of 31.41 k/mcl, hemoglobin of 7 g/dl, and platelets of 13 k/mcl. Blood urea nitrogen (BUN) was 15mg/dl, creatinine was 1.5mg/dl, Ca+ was 13mg/dl, Na+ was 132mmol/L, and K+ was 4mmol/L. He tested negative for COVID-19, and his liver function test showed alkaline phosphatase (ALP) of 203 unit/L, alanine transaminase (ALT) of 96 unit/L, aspartate aminotransferase (AST) of 140 unit/L, total protein of 11.4g/dl, and albumin of 3.2d/dl.

Given hypercalcemia, renal insufficiency, and gammopathy, serum free light chain, immunoglobin electrophoresis, and immunofixation were obtained and revealed igG Lambda monoclonal gammopathy at 4030mg/dl and a decrease of IgA and IgM.

**Figure 1:**
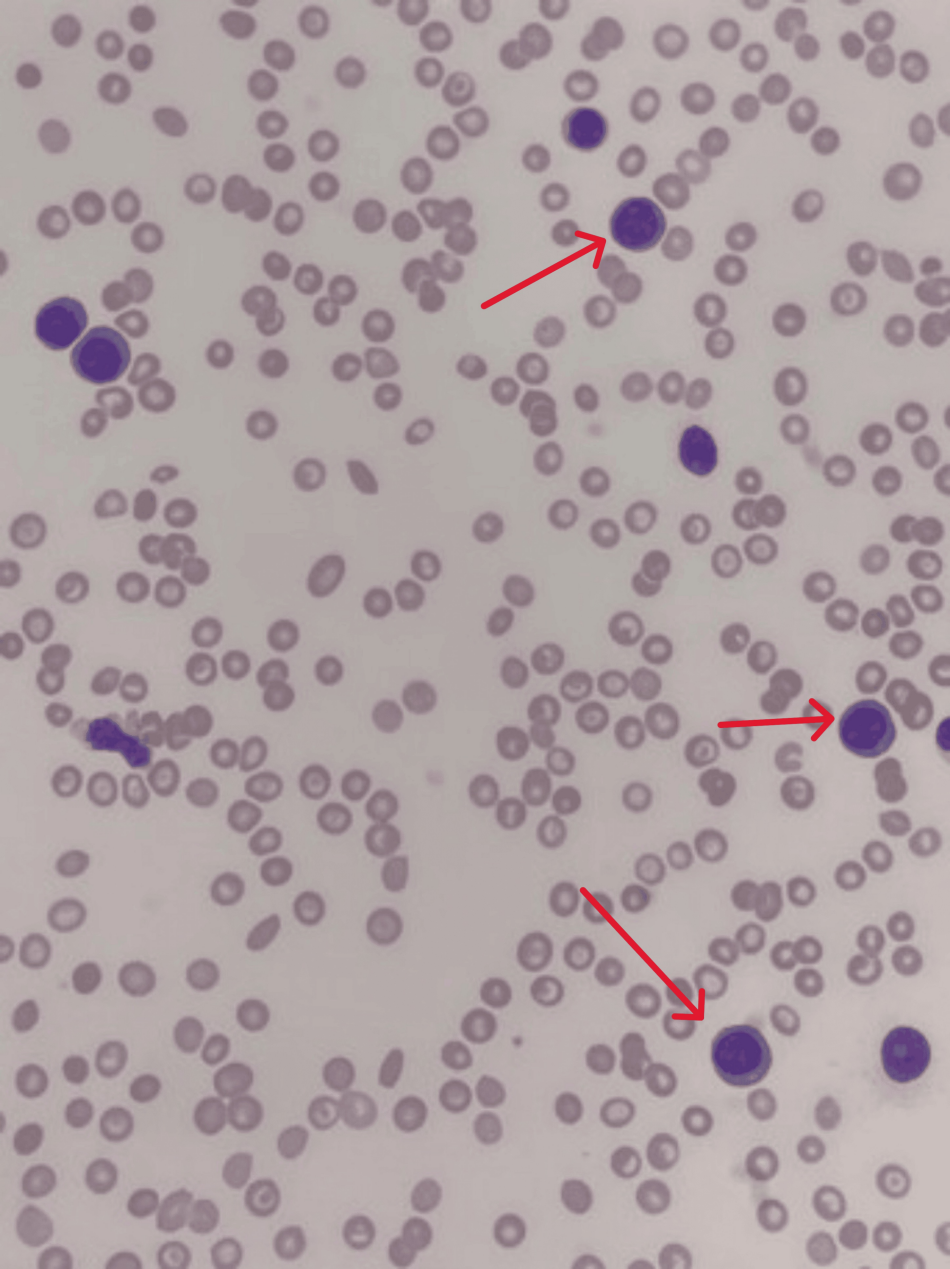
Peripheral blood smear Red arrows indicates malginant plasma cells

**Figure 2:**
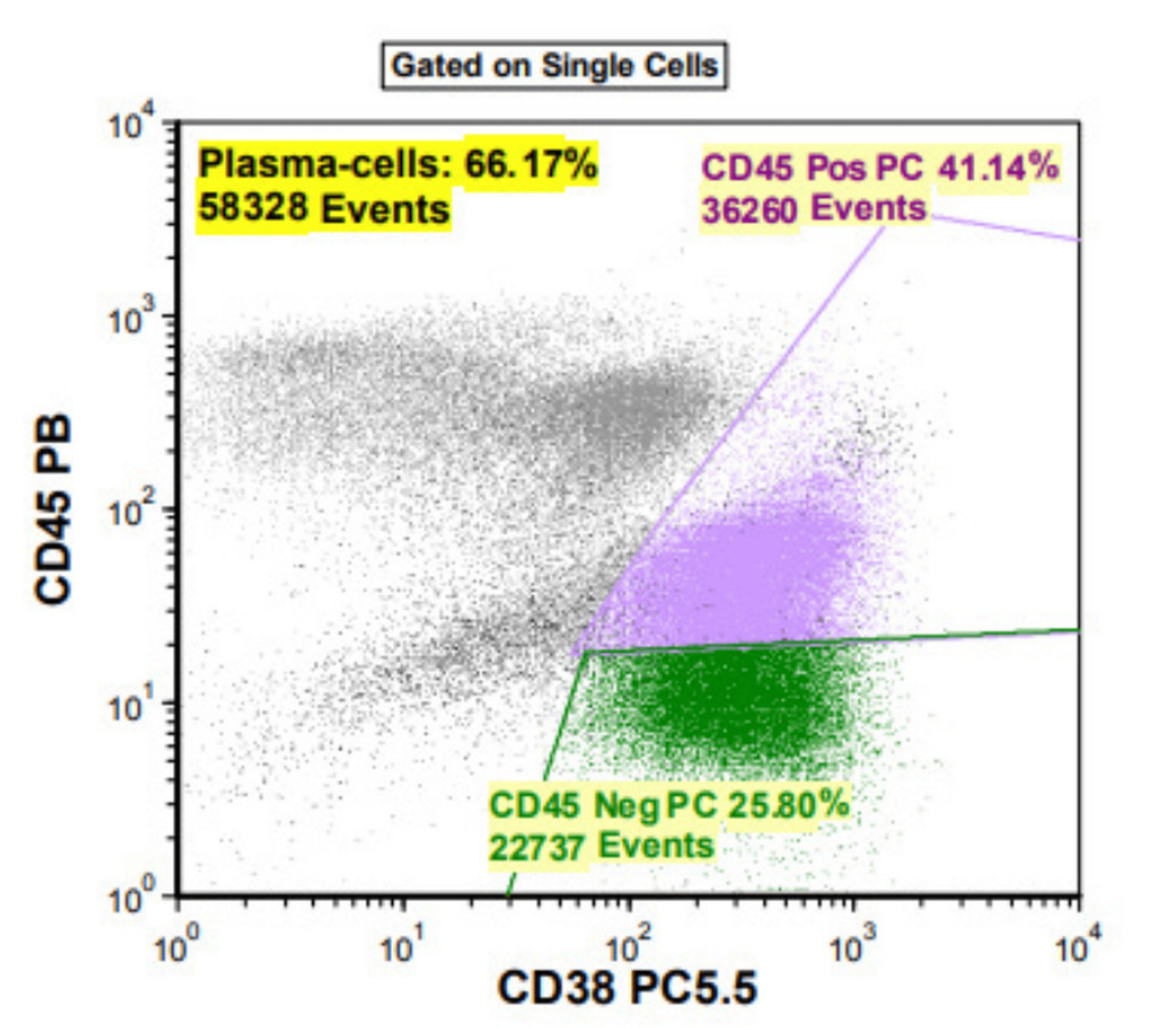
Flowcytometry

Peripheral smear (Figure 1) displayed a significant population of atypical mononuclear cells. Most have round or oval nuclei, medium-size nucleoli, and variable amounts of pale blue basophilic cytoplasm. Many had plasmacytoid features. Flow cytometry (Figure 2) Revealed a dense population of CD38+, CD45, and CD117+ cells in correlation of morphology; this was highly associated with plasma cell leukemia, with no finding associated with acute myeloid leukemia or non-Hodgkin lymphoma.

**Figure 3:**
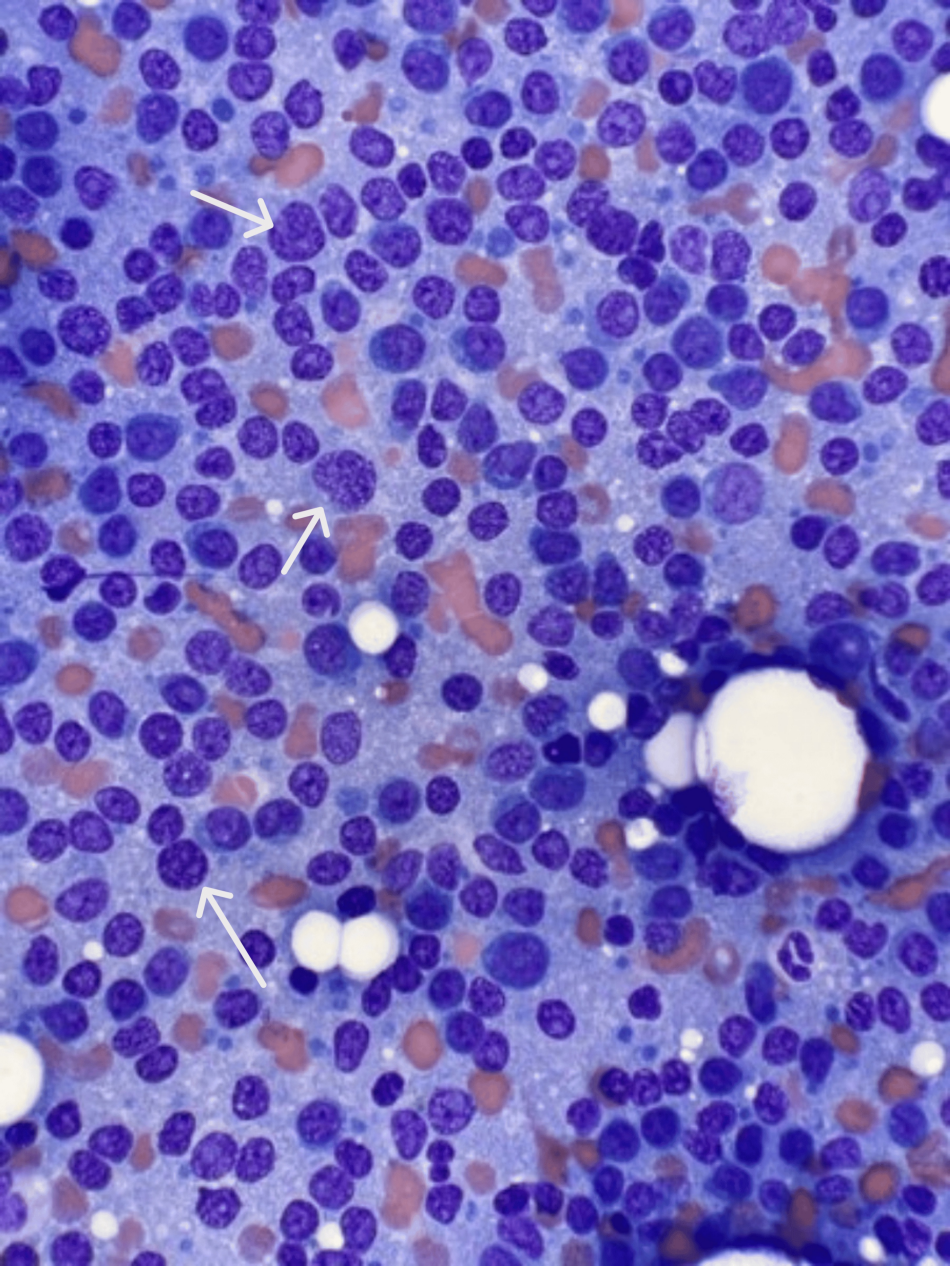
Bone marrow aspiration microscopic inspection White arrows indicate malignant cells

Subsequent bone marrow biopsy revealed plasma cell cytogenetics testing revealed loss 1p, monosomy 13, and deletions of chromosome 17p, resulting in the loss of TP53 and gain of chromosomes 5, 9, and 11. Fluorescent in situ hybridization showed the following abnormalities: monosomy 13, homozygous deletion of 1p, and TP53 deletion. All are associated with poor prognosis. Figure 3 reveals a microscopic examination.

## Discussion

PCL is an exceedingly rare variant of myeloma with unique circulating plasma cells. The course is aggressive, with short remission and survival [5]. A very prognostic finding is the presence of plasma cells circulating, with levels 5-20% associated with poor survival irrespective of age [6].

PCL arises in two ways, either a progression of underlying myeloma or without any previous disorder (de novo) [1] (Table 1).

**Table 1:** Table differentiaiting between primary and secondary plasma cell leukemia

	Primary cell leukemia	Secondary cell leukemia
Incidence	60=70%	30-40% increasing incidence due to improved survival of patients with MM
Clinical features	Hypercalcemia, lytic bone lesions, bone pain. Anemia, Leukocytosis, anemia, thrombocytopenia, infections, and hepatomegaly and splenomegaly	40-50% of patients will have already been diagnosed with MM
Clinical features	TP53 has been deleted in almost half of primary PCL. Primary PCL include the loss of chromosomes 16 (80%), 7 (11%), and X (25%), and trisomy of chromosome 8(43%)	TP53 has been detected in three-quarters of secondary PCL
Treatment	Determine eligibility for autologous hematopoietic cell transplantation. Multidrug induction regimen that includes bortezomib	Determine eligibility for autologous hematopoietic cell transplantation. Multidrug induction regimen that includes Bortezomib
Prognosis	Poor	Worse

To establish PCL, the percentage of plasma cells should be >5% to confirm PCL [7]. Table 2 summarizes the diagnostic evaluation.

**Table 2:** Diagnostic features of plasma cell leukemia

	Diagnostic evaluation
Peripheral smear	The proportion of plasma cells detected should be > 5%. Immature cells featuring dispersed nuclear chromatin, and an elevated nuclear-to-cytoplasm ratio.
Immunophenotype	CD20, CD23, CD28, CD44 Are more frequently expressed [2].
Bone marrow aspiration	Bone marrow infiltration is extensive, with plasma cells expressing a high proliferative index and anaplastic morphology.
Protein electrophoresis and immunofixation	Monoclonal Immunoglobulin production, with IgG being the most common.

A study published in November 2022 and reported in the Journal of Clinical Oncology by Jelinek et al. revealed that a level of 2% of circulating plasma cells (CTCs) would indicate PCL-like multiple myeloma [4]. CTCs look like marrow plasma cells; however, they don't have CD56 expression, while MM cells with PCL have more incidence of monosomy 13, Del 17p, and defects in chromosomes 1, 1q21, and others. No single abnormality that defines PCL. Cases showing multiple cytogenetics features, with most being known to be present in high-risk MM [8].

Treatment must start right away from diagnosis, with a management plan determined by eligibility for autologous hematopoietic cell transplantation. In case of eligibility, three to six cycles of induction therapy followed by autologous hematocrit (HCT). Ineligible for HCT would require an 8-12 induction cycle until the disease progresses or limiting toxicity [9].

No agreement for induction therapy for PCL, and most experts recommend regimens that include bortezomib, a proteasome inhibitor [7]; although early data about carfilzomib are hopeful, using it as initial therapy is not prevalent [10, 11].

The overall survival (OS) is extremely grim and averaged four months, and this contrasts with MM, in which there has been a slight improvement in OS over time [12].

A case reported in BMJ showed a 39-year-old male who was previously healthy presented with a two-week history of persistent fever, lethargy, and back pain. He denied lower extremities weakness, bleeding tendencies, or night sweats. A further investigation, which included peripheral edema, showed rouleaux formation with a high number of circulating plasma cells (almost 45%), and immunophenotyping by flow cytometric analysis revealed a 49% cluster of plasma cells with CD20, CD38, CD138, and C-Lambda. Diagnosis was confirmed following a bone marrow biopsy, showing 90% neoplastic plasma cells [13].

Patients with PCL are not cured with treatment. Treatment alleviates symptoms, reverses cytopenia, and decreases end-organ damage, and is given with the overall goals of achieving and maintaining a response, improving quality of life, and prolonging overall survival (OS). There is often no better option with patients with relapse disease rather than referring them to well-structured, scientifically valid clinical trials [14].

## Conclusions

Plasma cell leukemia is an aggressive tumor commonly present in patients with multiple myeloma. It is defined by the presence of plasma cells in the peripheral blood. It can arise with no prior history of MM. It arises in a minority of patients with MM, with clues that help early identification including circulating plasma cells on peripheral smear, development of hepatosplenomegaly or pleural effusion, or elevated lactate dehydrogenase.

New diagnostic criteria for PCL lowered plasma cells required from 20% in peripheral blood to 5% as both patients had similar prognoses; this helps to intervene promptly for patients with this aggressive disease. While treatment is not curative, treatment aims to improve quality of life and extend survival. There is no standard of care, and the approach used by experts varies. Patients should be sent urgently to an expert to help identify eligibility for HCT. There is limited information to guide the treatment of recurrent or refractory PCL, and care is individualized. Referring patients to clinical trials is appropriate.
